# A Recombinant Avian Leukosis Virus Subgroup J for Directly Monitoring Viral Infection and the Selection of Neutralizing Antibodies

**DOI:** 10.1371/journal.pone.0115422

**Published:** 2014-12-18

**Authors:** Qi Wang, Xiaofei Li, Xiaolin Ji, Jingfei Wang, Nan Shen, Yulong Gao, Xiaole Qi, Yongqiang Wang, Honglei Gao, Shide Zhang, Xiaomei Wang

**Affiliations:** 1 Division of Avian Infectious Diseases, State Key Laboratory of Veterinary Biotechnology, Harbin Veterinary Research Institute, Chinese Academy of Agricultural Sciences, Harbin, 150001, China; 2 Centre for Animal Infectious Disease Diagnosis and Technical Services and State Key Laboratory of Veterinary Biotechnology, Harbin Veterinary Research Institute, Chinese Academy of Agricultural Sciences, Harbin, 150001, China; 3 Departments of Radiology, Second Affiliated Hospital, Harbin Medical University, Harbin, 150086, China; Institut National de la Santé et de la Recherche Médicale (INSERM), France

## Abstract

Avian leukosis virus subgroup J (ALV-J) has induced serious clinical outbreaks and has become a serious infectious disease of chickens in China. We describe here the creation of a recombinant ALV-J tagged with the enhanced green fluorescent protein (named rHPRS-103EGFP). We successfully utilize the rHPRS-103EGFP to visualize viral infection and for development of a simplified serum-neutralization test.

## Introduction

Avian leukosis virus subgroup J (ALV-J), a member of the genus *Alpharetrovirus* in the *Retroviridae* family, was first isolated in the United Kingdom in 1988 [1]. ALV-J is a serious pathogen in chickens worldwide as it causes neoplastic diseases and immunosuppression. The virus is transmitted vertically from dam to progeny through the embryonic stage. ALV-J also has major public-health implications because of its potential threat to humans who receive vaccines produced in eggs or chicken embryonic fibroblasts (e.g., vaccines to measles, mumps, and yellow-fever) [2].

An EGFP-transducing virus can be used in a simplified and effective serum-neutralization test to monitor viral infection [3]. Although the immune fluorescence neutralization test is the gold standard for detecting neutralizing antibodies against ALV-J [4], these tests are labor-intensive and time-consuming due to the necessary incubation and staining procedures. EGFP-labeled virus offers the convenience of detecting neutralizing antibodies (NAbs) directly in unfixed cells. In this study, we engineered a recombinant ALV-J encoding EGFP, visualized infection of this recombinant virus in cells using live-cell imaging, and tested the recombinant virus against anti-ALV-J NAbs in sera using high-throughput screening.

## Methods

### Ethics Statement

All of the field serum samples were collected from the brachial vein by standard venipuncture procedure with all necessary permits obtained for the described field study. The field study did not involve endangered or protected species. All other chicken serum samples were collected from the brachial vein by standard venipuncture procedures, approved by the Animal Welfare and Ethics Committee of Veterinary Research Institute (HVRI) of the Chinese Academy of Agricultural Sciences (CAAS). The animal Ethics Committee approval number is Heilongjiang-SYXK-2006–032.

### Production and analysis of viral clones

The full-length infectious proviral molecular clone of HPRS-103 was a generous gift from Professor Venugopal Nair (denoted as pHPRS-103). To generate the recombinant plasmid pHPRS-103EGFP, a CMV-EGFP expression cassette (amplified by PCR from the pEGFP-C1 vector (Clontech, Palo Alto, CA, USA) was introduced into restriction enzyme sites AseI and BsrDI between position 7146 and 7224 of pHPRS-103. Virus rescue and identification was as described previously [5]. Briefly, highly purified pHPRS-103 and pHPRS-103EGFP DNA was obtained using QIAGEN Plasmid Midi kits (Qiagen, Hilden, Germany) according to the manufacturer's instructions. Purified plasmid DNA (4µg) of pHPRS-103 and pHPRS-103EGFP was transfected into DF-1 cells using Lipofectamine 2000 (Invitrogen, Carlsbad, CA, USA) and the culture supernatant, containing virus, was harvested 48h later. The rescued viruses were named rHPRS-103 and rHPRS-103EGFP. 0.1ml of rHPRS-103 (10^2^ TCID_50_ ml^ -1^) and rHPRS-103EGFP (10^2^ TCID_50_ ml^−1^) was used to infect DF-1 cells in order to explore the EGFP fluorescence signal. DF-1 cells infected with virus were fixed with 4% paraformaldehyde in PBS for 30 min. The cells were then incubated with anti-p27 antibody for 2h followed by incubation with goat anti-mouse IgG (whole molecule)–TRITC antibody (Sigma, USA). Cells were stained with 4, 6-diamidino-2-phenylindole (DAPI) for 15 min and examined using a Leica SP2 confocal system (Leica Microsystems, Germany). Signal colocalization was analyzed with the program Colocalizer Pro (Colocalization Research Software, Boise, ID).

### The replication kinetics of the rescued viruses and reverse transcriptase (RT) assay

To determine the replication kinetics of rHPRS-103 and rHPRS-103EGFP, DF-1 cells were infected in 60mm diameter plates with approximately 0.1 ml of 10^2^/ml TCID_50_ rHPRS-103 and rHPRS-103EGFP, respectively. Infected cell cultures were harvested at various time points, and infectious progeny titer was determined as TCID_50_ per milliliter using the Reed-Muench formula directed by indirect immunofluorescence assay (IFA) using anti-p27 antibody and anti-mouse IgG (whole molecule)–TRITC antibody. Mean values and standard deviations from three independent experiments were calculated. RT activity was quantitated using the colorimetric Reverse Transcriptase Assay (Roche Applied Science, Indianapolis, Indiana). Briefly, a 96-well plate (Product number: 6005225, PerkinElmer, Waltham, MA, USA) seeded with DF-1 cells were infected with approximately 0.02 ml of 10^2^/ml TCID_50_ of rHPRS-103EGFP. Post-infection culture supernatants were collected daily for 6 days and RT activity was quantitated using the manufacturer's protocol.

### Electron microscopy and living cells imaging

The ultra-thin section of the rHPRS-103EGFP-infected DF-1 cells were examined by electron microscopy as described previously [6]. In immunogold experiments, DF-1 cells were fixed with 4% formaldehyde in PBS (pH 7.2) for 30 min on ice, followed by treatment with 8% formaldehyde in PBS for another 120 min on ice. Fixed cells from the culture flask were manually scraped off, infiltrated with 2.1 M sucrose in PBS, and frozen in liquid nitrogen.100-nm diameter cryosections were obtained with a Leica Ultracut UCT/EM FCS cryo-ultramicrotome at −100°C. Thawed cryosections were blocked with 1% milk powder−0.5% bovine serum albumin in PBS and incubated with rabbit anti-GFP (1∶200; Torrey Pines Biolabs) in blocking buffer for 60 min. After washing with blocking buffer, bound antibodies were detected with goat anti-rabbit IgG coupled to Nanogold (Nanoprobes) or ultrasmall gold (Aurion). Silver enhancement was performed as described by Danscher and by Stierhof et al [7], [8]. Final embedding was done in 2% methyl cellulose containing 0.3% uranyl acetate. Ultrathin sections were viewed in a LEO 906 transmission electron microscope.

For long-term live cell imaging, DF-1 cells were plated on 35-mm imaging dishes overnight before infection with 0.1ml rHPRS-103EGFP (10^2^ TCID_50_ ml ^−1^). Imaging was carried out 2 h after infection. For time-lapse movies, EGFP channels were imaged every 30min. All time-lapse movies were edited using Volocity software (Perkin Elmer, Waltham, MA, USA) and saved for presentation in mp4 format.

### Cell culture and Viral neutralization tests

DF-1 cells were maintained in Gibco Dulbecco's Modified Eagle Medium (DMEM, Invitrogen, Carlsbad, CA, USA) supplemented with 10% FBS at 37°C under 5% CO_2_. Viral neutralization tests were carried out on DF-1 cells through a microneutralizationassay [9] using subgroup-J-specific antisera and 0.1 ml per plate of 10^2^/ml TCID_50_ virus. Briefly, DF-1 cells were added to each well in a 96-well cell plate (Product number: 6005225, PerkinElmer, Waltham, MA, USA). The plates were placed in a 37°C CO_2_ incubator for 24h. Serial dilution of heat-inactivated serum was mixed with virus for 2h in a 37°C CO_2_ incubator. The cell culture media in the 96-well plate was carefully aspirated and replaced with the serum and virus mixture. The plates were briefly rocked and placed in a 37°C CO_2_ incubator for 2 h. The inoculant was then aspirated, and each well received 100µl DMEM with 2% FBS. The plate was placed in a 37°C CO_2_ incubator for 6 days. Fluorescence was detected with the Opera QEHS high-content screening platform (PerkinElmer, Waltham, MA, USA). An Opera QEHS system (PerkinElmer, Waltham, MA) was used for high-throughput image acquisition. 5 imaging fields per well were acquired with a 10× objective in the Blue (Hoechst 33342) and Green (Alexa488) channels on a single Z-plane. Images were imported into Columbus 2.3 database (PerkinElmer) and analyzed with Acapella 2.7 (PerkinElmer). To measure the EGFP fluorescence/DAPI quantification, cell nuclei were first identified using the Hoechst33342 channel image, and EGFP was detected using the Alexa 488 channel image. The nuclei detection described above generated a first population of objects (Nuclei). All of the cellular attributes fromthe Nuclei population were then imported as sums. The percentage of EGFP fluorescence/DAPI was calculated as (Number of EGFP objects)/(Number of Nuclei objects)*100. Data were analyzed and the summary of the identified EGFP-positive cells.

## Results

### Rescue of recombinant ALV-J expressing EGFP

**Figure 1:**
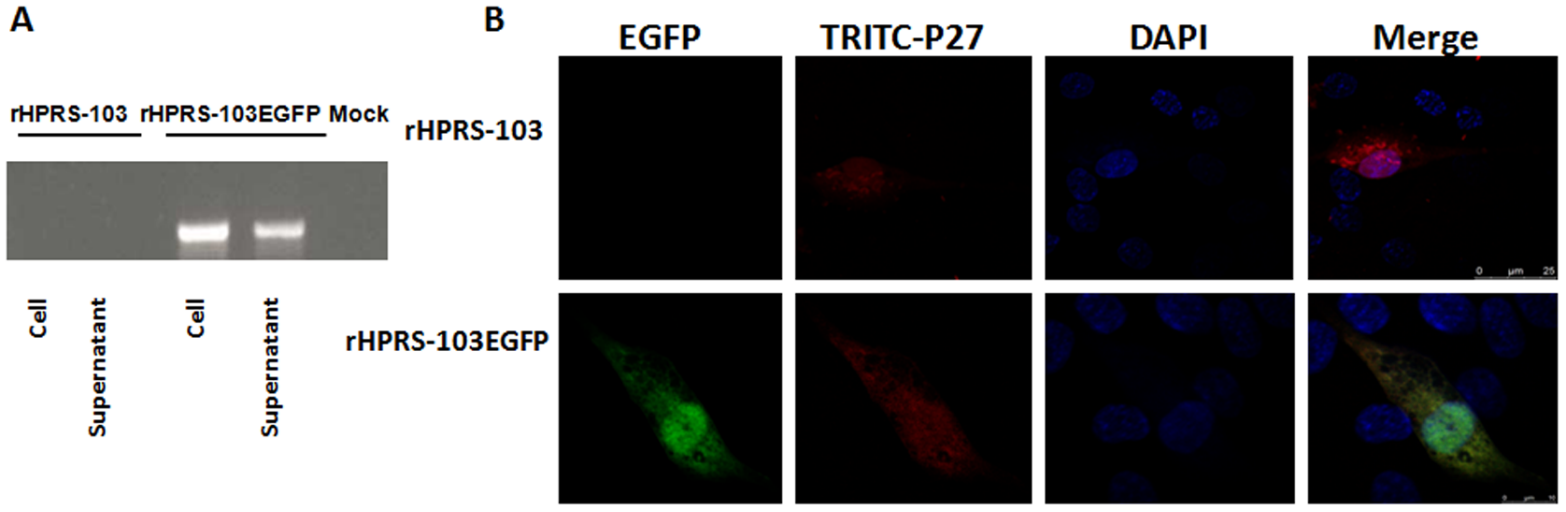
Generation of infectious ALV-J reporter viruses. A, Detection of recombinant genomes in supernatants and cell lysates of rHPRS-103EGFP-infected DF-1 cells by RT-PCR. RT-PCR was conducted using primers that bind to the EGFP ORF. B, Fluorescence microscopy of rALV-J-EGFP-infected cells. Cells were stained with a monoclonal antibody reactive to the ALV-J p27 protein, and EGFP expression was examined. Cells were fixed at 48 h postinfection and subjected to indirect immunofluorescence to detect p27 protein (red) and EGFP autofluorescence. The position of the nucleus is indicatedby DAPI (blue) staining in the merged image.

Successful rescue of rHPRS-103EGFP containing the EGFP gene was confirmed by RT-PCR and through immunofluorescent detection of EGFP expression in infected cells. Stable integration of the EGFP gene into the viral genome was verified by amplification of the 798 bp EGFP PCR fragment obtained from cDNA of rHPRS-103EGFP-infected cells (Fig. 1A). Sanger sequencing analysis confirmed the EGFP gene target. The expression of EGFP in living DF-1 cells was analyzed by fluorescence microscopy. The rHPRS-103EGFP-infected cells were examined by indirect immunofluorescence using anti-p27 antiserum recognizing the viral proteins. EGFP autofluorescence was assessed in parallel. Cytoplasmic EGFP aggregates colocalized with ALV-J-induced inclusions (Fig. 1B). These data show that rHPRS-103EGFP productively infects susceptible cells and can be used as a sensitive marker to visualize viral infection.

### Correlation of EGFP levels and rHPRS-103EGFP expression *in vitro*

**Figure 2:**
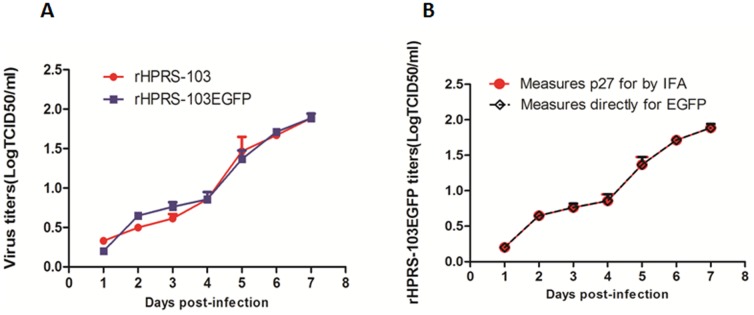
Replication of rHPRS-103EGFP and rHPRS-103. A, Growth kinetics assay of rHPRS-103EGFP and rHPRS-103. B, The growth kinetics of rHPRS-103EGFP was measured by p27 expression using IFA while the fluorescence is a direct measure of EGFP-positive cells.

To determine whether exogenous gene insertion affects recombinant virus replication, growth curves of rHPRS-103EGFP and rHPRS-103 were determined in DF-1 cells. No discernible difference in growth rate or maximum titer between the two viruses was observed (Fig. 2A). To further compare the sensitivity of virus infection between the EGFP expression and viral protein expression, the rHPRS-103EGFP growth curve was re-titrated by an additional two methods. These include 1) direct measurement of EGFP protein expression levels and 2) an IFA test for viral p27 expression. As shown in Fig. 2B, the rHPRS-103EGFP growth curves were similar using these two methods, which suggests that the EGFP expression and viral protein expression of rHPRS-103EGFP exhibit equal sensitivity of detection.

**Figure 3:**
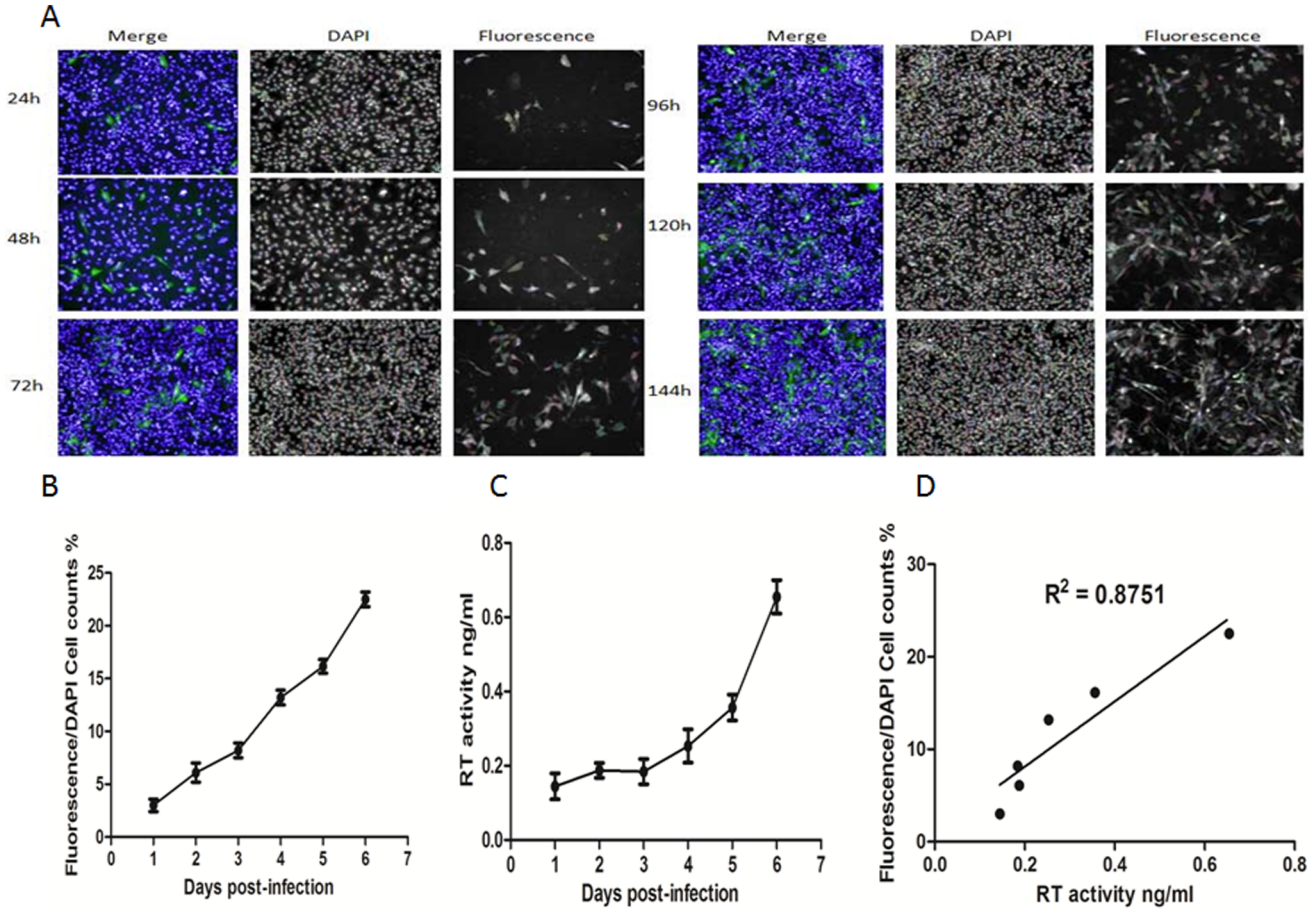
Characterization of rHPRS-103EGFP and the correlation between fluorescence levels and ALV-J replication. A, Time-lapse fluorescence microscopy for the spread of rHPRS-103EGFP. DF-1 cells were infected with HPRS-103EGFP and cells fixed and stained with DAPI every 24 h for a period of 6 days. B, The percentage of EGFP-positive cells shown in A. C, During the time-lapse fluorescence microscopy of rHPRS-103EGFP, the supernatants of rHPRS-103EGFP-infected cells were examined for RT activity. D, Correlation between the RT activity and the EGFP positive cells. The viral titers harvested at different intervals were calculated and expressed as TCID_50_ per milliliter. The RT activity was quantitated using a colorimetric Reverse Transcriptase Assay (see Methods). The standard deviations (error bars) from 3 independent experiments are shown.

To investigate the relationship between EGFP expression levels and viral replication, DF-1 cells were infected with rHPRS-103EGFP. The percentage of EGFP-positive cells was measured at the indicated time points using a high-throughput screening assay (Fig. 3A and 3B). In parallel, reverse transcriptase (RT) activity levels were assayed at each time point. As expected, a time-dependent increase in the percentage of EGFP-positive cells was associated with the number of rHPRS-103EGFP-infected cells (Fig. 3A and 3B). Furthermore, viral RT levels in the supernatants were detected (Fig. 3C) and were highly correlated with % EGFP-positive cells up to 144 h post-infection (Fig. 3D).

### Intracellular EGFP expression represents rHPRS-103EGFP infection but not virion particles

**Figure 4:**
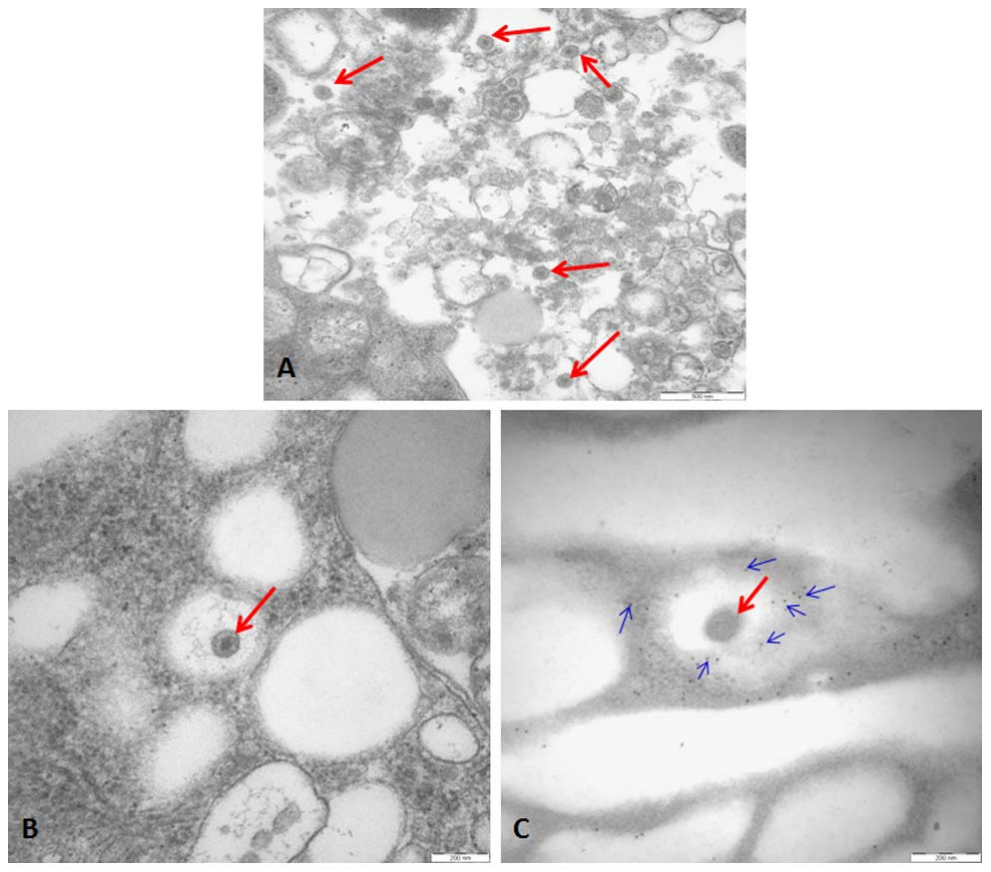
Microscopic analysis of rHPRS-103EGFP particles. A, Electron microscopy of ultrathin sections of rHPRS-103EGFP-infected DF-1 cells revealed the virion. Scale bar  =  500 nm. B, The zoomed-in images of individual virion in electron microscopy of ultrathin sections. Scale bar  =  200 nm. C, The individual virion in immunogold labeling on ultrathin cryosections. Scale bar  =  200 nm. The red arrows point to virion. The blue arrows point to EGFP.

To determine whether the EGFP signal actually represents virion particles, we used immunogold labeling of EGFP and subsequent microscopy. DF-1 cells were infected with rHPRS-103EGFP. Ultrathin cryosections were labeled with anti-EGFP antibodies and silver-enhanced ultrasmall gold and were analyzed by electron microscopy. As shown in Fig. 4, this ultrastructural analysis demonstrated that EGFP is not a component of a single virion particle.

We next examined the infection of rHPRS-103EGFP in cell culture by live-cell imaging. DF-1 cells were infected with rHPRS-103EGFP and the cell monolayer was analyzed for EGFP fluorescence by taking a series of photomicrographs. Photomicrographs from five different positions were captured every half hour for a period of 6 days. We observed that an increasing number of cells were infected with rHPRS-103EGFP by using live-cell imaging (S1 Movie). The presence of green fluorescence within a previously naïve cell suggests that the cell was infected with rHPRS-103EGFP and the EGFP coding sequence was expressed correctly. In summary, these data demonstrate that green fluorescence expression, which remains diffusely intracellular, represent the viral infection and does not specifically track to single virion particles.

### ALV-J neutralization test using rHPRS-103EGFP

**Figure 5:**
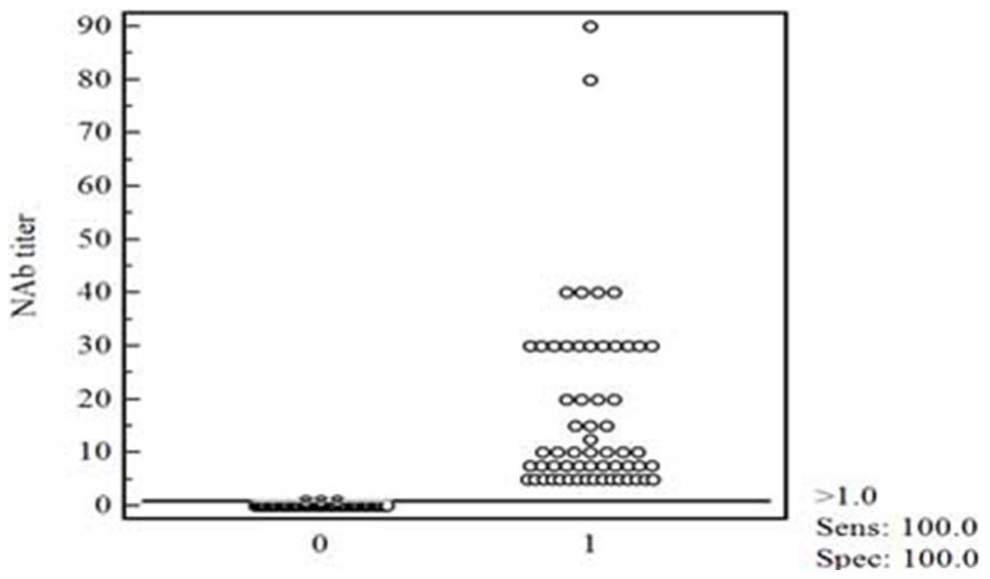
Interactive dot diagram for the neutralization test using rHPRS-103EGFP. Interactive dot diagram of NAbs titers from samples (n = 110) detected by NT-EGFP relative to the samples in parallel detected by NIT. The cutoff value of NT-EGFP was 1. The NAb titer is the reciprocal of the highest dilution. “0” stands for the negative results detected by NIT and “1” stands for the positive results detected by NIT.

EGFP expression from rHPRS-103EGFP was easily observed in live cells by fluorescence microscopy. To determine whether EGFP expression from rHPRS-103EGFP could be used to in a neutralizing antibody assay against ALV-J, experimental sera from ALV-J-infected chickens (n = 30), field serum samples (n = 50), ALV-J-negative sera (n = 30) originating from healthy SPF chickens, and chicken antisera against IBDV, ARV, ALV-A, ALV-B, and MDV (n = 3 for each) from our Institute were collected. Specificity and sensitivity of the neutralization test was evaluated based on EGFP expression from rHPRS-103EGFP (NT-EGFP). All sera were tested in parallel by neutralization immunofluorescence tests (NIT) using rHPRS-103 and the subgroup-specific antibody JE-9. Receiver operating characteristic (ROC) analysis was performed with MedCalc software to determine the cutoff value of NT-EGFP and the relative specificity and sensitivity between NT-EGFP and NIT. The ROC analysis showed that the cutoff value of NT-EGFP was 1, and the relative sensitivity and specificity of NT-EGFP were both 100% compared to NIT (Fig. 5). Next, we examined whether the NAbs titers determined by NT-EGFP were consistent with those determined by NIT. For the experimental sera, the agreement between NT-EGFP and NIT was 93.3% (28/30). For the field sera, the accordance between NT-EGFP and NIT was 94% (47/50) (Table 1). Antisera against non-ALV-J chicken viruses, including IBDV,ARV,ALV-A,ALV-B, and MDV, was used to evaluate NT-EGFP cross-reactivity. These sera all scored negative (with NAbs titers <1) in NT-EGFP indicating the high specificity of NT-EGFP.

**Table 1:** Agreement between ALV-J-specific neutralizing antibody titers determined by NIT and NT-EGFP.

Sera		No. of samples		Agreement
		Identical titers	Different titers	
Experimental sera	Positive	12	2	93.3%
	Negative	16	0	
Field sera	Positive	42	3	94%
	Negative	5	0	

## Discussion

Here we report the generation of a recombinant ALV-J virus expressing EGFP inserted between positions 7146 and 7224 of the HPRS-103 genome. We chose this EGFP insertion location to allow the rescue of chimeric clones in the 3′untranslated region [5]. The 3′untranslated region, which contains rTM, DR-1, the E element, and the 3′LTR, is very important to the pathogenicity of ALV [5], [10], [11], [12]. Although similar replication of rHPRS-103EGFP and rHPRS-103 was observed in cell culture, insertion of EGFP into 3′untranslated region of ALV-J may influence the pathogenic potential of ALV-J. Dana Kucerová et al. has also show the ability to rescue a recombinant ALV-J expressing EGFP called RCAS(J)GFP. The env gene of RCAS(A)GFP was replaced with the complete env gene from the cloned HPRS-103[13]. The titer of RCAS(J)GFP produced in DF-1cells was able to reach 10^6^ IU per ml. This titer is higher than rHPRS-103EGFP rescued in our study, which may be attributed to the fact that gag, pol, and LTR coding regions of RCAS(A)GFP are contained within RCAS(J)GFP but not HPRS-103. Gag, pol and LTR coding regions of RCAS(A)GFP are not completely identical within HPRS-103. Furthermore, gag, pol and LTR regions are all involved in the replication of ALV within avian cells [14], [15]. Hence, the titer of RCAS(J)GFP and rHPRS-103EGFP are different although they both are ALV-J expressing EGFP molecular clones.

Immunogold labeling EGFP showed that the green fluorescence expression in rHPRS-103EGFP infected cells do not represent single virion particles because the EGFP protein is not a structural or enzymatic component of rHPRS-103EGFP virus (Fig. 4). To successfully track a single virus particle, the fluorescence protein or small molecular dyes would have to be fused with a viral protein [16]. In our study, the EGFP gene was inserted into the 3′UTR and this region does not encode a viral protein. However, using the live-cell imaging, we could observe the infection of rHPRS-103EGFP directly in cell culture (S1 Movie). Some retroviruses, such as human immunodeficiency virus (HIV) and the murine leukemia virus (MLV), have two transmission routes in cell culture: conventional cell-free retroviral infection and direct cell-to-cell transmission [17], [18], [19]. We observed that uninfected neighboring cells of the infected EGFP-expressing cells became infected, which suggests that ALV-J may also exhibit direct cell-to-cell transmission. Additional analyses and characterization of a potential virological synapse are needed to substantiate this hypothesis. Furthermore, our study shows that it is much easier to determine the infection of rHPRS-103EGFP by directly observing EGFP expression. The standard assays for determining virus infection is to detect the viral protein expression and it is a more complicated and time-consuming process compared to direct observation of EGFP expression.

In this study, we also demonstrated the feasibility of a new ALV-J neutralizing antibody assay using rHPRS-103EGFP. Compared to the standard virus neutralization assay using subgroup-J-specific antisera, this new assay is faster and does not require excessive washing. Based on high-throughput screening, rHPRS-103EGFP could be applied in a visual screen to identify neutralizing antibodies against ALV-J. Four classes of avian leukosis viral infections are recognized in mature chickens: 1) no viremia, no antibody [V-A-]; 2) no viremia, with antibody [V-A+]; 3) viremia, with antibody [V+A+]; and 4)viremia, no antibody [V+A-][20]. In the fourth condition, the viremia may influence serum-neutralization tests based on the traditional method; however, the neutralizing antibody assay using rHPRS-103EGFP could overcome this influence of viremia in the samples and more precisely reveal the neutralizing antibody condition. Furthermore, EGFP inserted into the 3′UTR of ALV-J and the env gene of rHPRS-103EGFP could be exchanged. As the env gene of ALV determines the subgroup specificity and neutralization [9], viruses constructed in this study could be used to determine neutralizing antibodies for other subgroups.

Overall, this study describes the generation and characterization of an EGFP-containing ALV-J, which will be a useful tool for the image-based screening of viral infection and the detection of neutralizing antibodies.

## Supporting Information

S1 Movie The infection of rHPRS-103EGFP in cell culture by live-cell imaging.DF-1 cells were infected with rHPRS-103EGFP and then photomicrographs of five different positions were captured every 30min using a 20 × magnifying objective. Only one representative view of the five was shown in the movie. The red arrow shows a cell infected with rHPRS-103EGFP followed by subsequent infection of its neighboring cell, upon rHPRS-103EGFP infection and EGFP expression.
(MP4)
