# Correction: A Recombinant Avian Leukosis Virus Subgroup J for Directly Monitoring Viral Infection and the Selection of Neutralizing Antibodies

**DOI:** 10.1371/journal.pone.0121770

**Published:** 2015-04-02

**Authors:** The PLOS ONE Staff

There is an error in the affiliation for the 11^th^ author. Xiaomei Wang should have additional affiliation #4 Jiangsu Co-innovation Center for Prevention and Control of Important Animal Infectious Disease and Zoonoses, Yangzhou, 225009, China.
